# Intracranial Arterial Dolichoectasia

**DOI:** 10.3389/fneur.2017.00344

**Published:** 2017-07-17

**Authors:** Victor J. Del Brutto, Jorge G. Ortiz, José Biller

**Affiliations:** ^1^Department of Neurology, Pritzker School of Medicine, University of Chicago, Chicago, IL, United States; ^2^Department of Neurology, Stritch School of Medicine, Loyola University Chicago, Maywood, IL, United States

**Keywords:** dolichoectasia, ectasia, dolichosis, dilatative arteriopathy, intracranial arterial dolichoectasia

## Abstract

An increased diameter (ectasis) and/or long and tortuous course (dolichosis) of at least one cerebral artery define intracranial arterial dolichoectasia (IADE). IADE could be detected incidentally or may give rise to an array of neurological complications including ischemic stroke, intracranial hemorrhage, or compression of surrounding neural structures. The basilar artery is preferentially affected and has been studied in more detail, mainly due to the presence of accepted diagnostic criteria proposed by Smoker and colleagues in 1986 (1). Criteria for the diagnoses of dolichoectasia in other cerebral arteries have been suggested. However, they lack validation across studies. The prevalence of IADE is approximately 0.08–6.5% in the general population, while in patients with stroke, the prevalence ranges from 3 to 17%. Variations among case series depend on the characteristics of the studied population, diagnostic tests used, and diagnostic criteria applied. In rare instances, an underlying hereditary condition, connective tissue disorder, or infection predispose to the development of IADE. However, most cases are sporadic and associated with traditional vascular risk factors including advanced age, male gender, and arterial hypertension. The link between this dilative arteriopathy and other vascular abnormalities, such as abdominal aortic aneurysm, coronary artery ectasia, and cerebral small vessel disease, suggests the underlying diffuse vascular process. Further understanding is needed on the physiopathology of IADE and how to prevent its progression and clinical complications.

## Introduction

**Figure 1:**
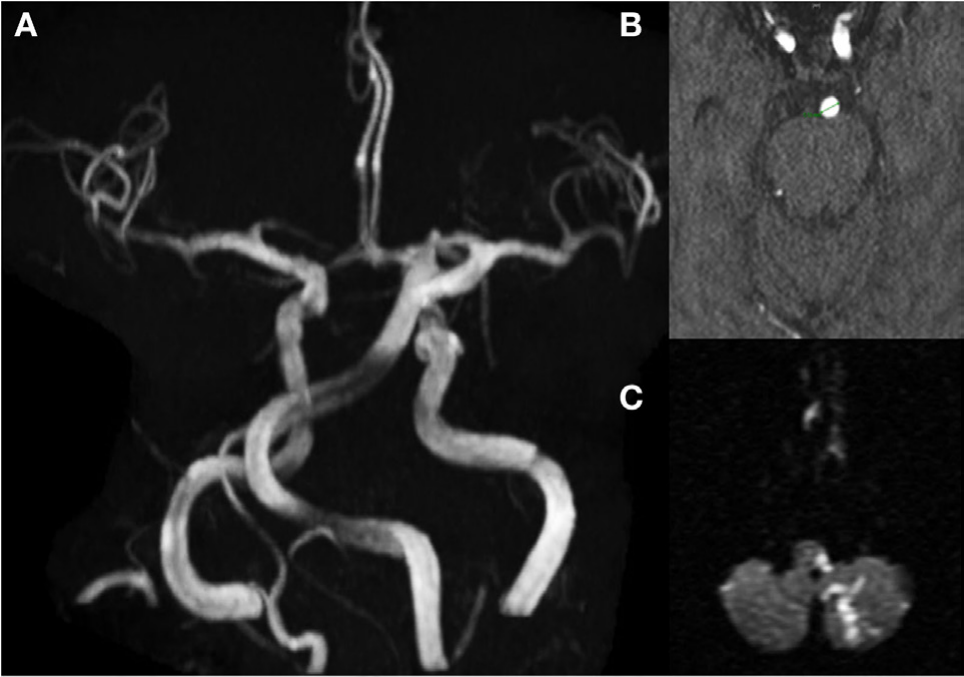
MRI/A brain of a 67-year-old man presenting with left lateral medullary syndrome, and left posterior inferior cerebellar artery (PICA) territory infarct; **(A)** MRA brain showing elongated and tortuous basilar artery (BA); **(B)** axial cut showing increased BA diameters (5.5 mm); **(C)** diffusion-weight restricted lesion on left PICA territory.

Intracranial arterial dolichoectasia (IADE) is an unusual arteriopathy characterized by abnormal elongation, tortuosity, and dilation of the cerebral arteries (2) (Figure 1). Different from the endothelial injury and plaque formation characteristic of atherosclerosis, the tunica media of the arterial wall in patients with IADE is affected by disruption of the internal elastic lamina, atrophy of the muscle layer, and hyalinization of connective tissue, leading to abnormal dilation of the affected blood vessel (3–5). Both, the anterior and posterior cerebral circulation could be affected. However, the basilar artery (BA) is by far the most common affected vessel (6, 7). The terms IADE, vertebrobasilar dolichoectasia (VBD), and BA dolichoectasia (BADE) have been used interchangeably in the literature. This might lead to some confusion, as carotid artery ectasia also occurs (2, 8).

Intracranial arterial dolichoectasia may be complicated by brain infarction, cranial nerves or brainstem compression, hydrocephalus, intracranial hemorrhage, or death (9–11). IADE has been associated with traditional cerebrovascular risk factors such as advanced age, male gender, arterial hypertension, and history of coronary artery disease (3, 12–14). IADE has also been associated with other vascular abnormalities such as abdominal aortic aneurysms (4, 11, 15), intracranial saccular aneurysms (11, 16, 17), and coronary artery disease with or without coronary artery ectasia (18, 19). In addition, a link between IADE and markers of small vessel disease (SVD) has suggested an unidentified common vascular process (7, 20, 21).

Although little is known about its prognosis, natural course, and how to prevent its complications, IADE is not a benign condition. The lack of validated quantitative criteria has made the study of IADE extremely challenging (1, 12). Having standard definitions is imperative to identify high-risk patients in clinical practice, as well as to conduct meaningful and validated research. In this article, we provide a review of available concepts and a discussion of the definition of IADE as well as the prevalence, risk factors, and known complications of this intriguing intracranial arteriopathy.

## Definition of IADE

The etymology of the word dolichoectasia comes from the Greek *dolikhós* that means long, and *ektasis* that literally means distention of a tubular structure (22). There is a spectrum between the normal variations on diameter and tortuosity of cerebral blood vessels and massive aneurysmal dilations leading to serious clinical complications (10, 11). Different terms such as mega-artery, mega dolichoartery, fusiform aneurysm, cirsoid aneurysm, serpentiform aneurysm, atherosclerotic aneurysm, and dilatative arteriopathy have been used to describe the abnormal distention and elongation of intracranial arteries (2, 3, 12, 22, 23). Mega-artery and mega dolichoartery represent expressions used for gigantic widening of intracranial vessels causing mass effect and pertained to the most severe forms of the disease (24, 25). The term aneurysm denotes a localized widen-out due to weakening of the blood vessel wall (26). Intracranial aneurysms could be saccular or non-saccular according to their shape (26, 27). Fusiform aneurysm is a form of non-saccular arterial dilation characterized by circumferential ballooning of the entire vessel wall for a short segment (27). The concept of fusiform aneurysm overlaps with the definition of dolichoectasia. However, the later also incorporates the elongation and tortuosity (dolichosis) of an artery in addition to its segmental widening (27, 28). Atherosclerotic aneurysm is a misnomer, as arterial dolichoectasia is a distinct arteriopathy from atherosclerosis. However, they do share similar risk factors (3, 7, 21). Because increased diameter (ectasis) is the most important feature of IADE, dilatative arteriopathy has been used as an alternative name (2).

Intracranial arterial dolichoectasia describes the presence of at least one ectatic and/or enlarged artery in the cerebral vasculature (3). Within the brain, the vertebrobasilar system is preferentially affected (6, 7) and probably easier to evaluate on brain imaging than the anterior circulation. For these reasons, VBD and BADE have been preferentially studied and better defined in the literature. Pico and collaborators (3) proposed IADE as a more accurate term to avoid overlooking for anterior circulation pathology. More recently, diffuse IADE, defined as the involvement of two or more cerebral blood vessels, has been suggested as a distinct vascular presentation with a poorer natural history including higher rates of aneurysmal-related death (29).

To determine ectasia, previous investigators have suggested the use of two SD cutoffs based on the maximum diameter of the affected blood vessel (1, 6–8). Smoker and colleagues (1) recommended a cutoff of 4.5 mm diameter at the level of the mid pons to define BA ectasis based on their analysis of 126 CT scans of individuals without underlying brain injury (age ranging from 4 to 85 years). This diameter cutoff has been well accepted (25, 30–35). Gutierrez and collaborators (6) used an arterial diameter of ≥2 SD adjusted for the total cranial volume to define BA ectasis in a large multiethnic cohort and found major variations in the reported frequency when compared to the use of Smoker et al. criteria, visual assessment, and BA volume (4.3 vs 2.9 vs 18.8 vs 2.4%, respectively). For vessels other than the BA, Passero and Rossi have also suggested diameter cutoffs for the internal carotid (≥7 mm), middle cerebral artery (≥4 mm), and vertebral artery (≥4 mm) to indicate ectasia (10). The evidence of increased clinical complications associated with higher degree of ectasis supports definitions based on arterial diameter (6, 10, 11, 36). For research purposes, the use of arterial diameter as a continuous data rather than a binary categorization would provide more reliable information about the clinical implications of IADE.

Dolichosis refers to the abnormal elongation and tortuosity of an artery. Anatomical landmarks have been used to determine an irregular arterial course. A BA bifurcation at the floor of the third ventricle (above the suprasellar cistern) or a course lateral to the margin of the clivus is considered abnormal (1). The vertebral artery has been considered abnormal if a deviation of >10 mm from the shortest expected course or a length >23.5 mm exists (31). In order to determine dolichosis of the supraclinoid segment of the internal carotid artery, anterior cerebral artery, middle cerebral artery, and posterior cerebral artery, Gutierrez et al. have proposed a visual assessment based on the compression of surrounding structures and the tortuosity of the vessel when compared with the contralateral artery (37).

As noticed, an objective definition of IADE has been limited due to the lack of established benchmarks. The development of validated criteria remains challenging due to the lack a gold standard test. Additional studies are needed to better define the cutoffs for pathological IADE in the population.

## Prevalence

Table 1 (6, 16, 22, 36, 38) illustrates the reported prevalence among stroke-free patients. Table 2 illustrates the reported prevalence among stroke patients (7, 14, 21, 39–42). One study among the general population considered all large intracranial arteries for the definition of IADE and found that approximately 19% of individuals 55 years or older had at least one dolichoectatic intracranial artery (6). In this study, the IADE definition was based on the arterial diameter adjusted for head size and involved a multiethnic cohort. However, similar results have not been reproduced by other groups (6). Other large series may have been biased to Asian populations (16, 38) or to hospitalized patients undergoing brain MRI for any reason (36). Further studies are needed to elucidate the prevalence of IADE in the general population.

**Table 1:** IADE prevalence in series confined to stroke-free patients.

Reference	Population studied (*n*)	Definition of dolichoectasia	Diagnostic method	Prevalence (%)
Gutierrez et al. (6)	Multiethnic cohort of stroke-free individuals >55 years old (NOMAS) (718)	TCV-adjusted arterial diameter ≥2 SD	MRA + automated software tool	IADE 18.8, BADE 4.3
Tanaka et al. (38)	Outpatients with atherosclerotic risk factors >40 years old (493)	BA diameter >4.5 mm	T2-weighted MRI, MRA	BADE 0.8.
Vasović et al. (22)	Autopsy studies (age range 0–95 years old) (216)	BA or VA outer diameter >4.3 mm, with or without deviation from the shortest expected course, and BA length >33 mm	Autopsy	VBD 6.5.
Ikeda et al. (16)	Working-class adults living in Tokyo aged 30–90 years old (7,345)	BA diameter >4.5 mm; VA diameter >4.0 mm	MRI, MRA	VBD 1.3
Wolfe et al. (36)	Patients with neuroimaging from a University Hospital cohort (1,440)	BA diameter >4.5 mm; BA length >29.5 mm or lateral deviation >10 mm; VA length >23.5 mm or lateral deviation >10 mm	MRA	VBD 4.4

*BA, basilar artery; BADE, basilar artery dolichoectasia; VA, vertebral artery; VBD, vertebrobasilar dolichoectasia; IADE, intracranial arterial dolichoectasia; TCV, total cranial volume*.

**Table 2:** IADE prevalence in series confined to stroke patients.

Reference	Population studied (*n*)	Definition of dolichoectasia	Diagnostic method	Prevalence (%)
Nakajima et al. (14)	Patients with lacunar strokes (SPS3 trial) (2,621)	BA diameter >4.5 mm; VA diameter >4.0 mm	MRA, CTA	VBD 7.6
Park et al. (39)	Patients with ischemic stroke or TIA (182)	BA diameter >4.5 mm, and either BA bifurcation above the suprasellar cistern or lateral to the margin of the clivus (dolichosis)	MRA	VBD 13.2
Nakamura et al. (40)	Patients with acute ischemic and hemorrhagic stroke (481)	BA diameter >4.5 mm; VA diameter >4.0 mm	MRI, MRA	VBD 7.7 (ischemic stroke 6.4; hemorrhagic stroke 12.1)
Pico et al. (21)	Autopsy of patients with ischemic or hemorrhagic stroke (381)	Enlargement and tortuosity by visual assessment on pathological examination	Autopsy	IADE 6.0
Pico et al. (7)	Caucasian patients with ischemic stroke proven by MRI (510)	Visual assessment	CT, CTA, MRI	IADE 12
Ince et al. (41)	Patients with ischemic stroke in the community (The Rochester Epidemiology Project) (387)	Visual assessment	CT, MRI	IADE 3.1
Bogousslavsky et al. (42)	Patients with posterior circulation stroke >45 years old (70)	Visual assessment	MRI, MRA	VBD 17.1

*BA, basilar artery; BADE, basilar artery dolichoectasia; VA, vertebral artery; VBD, vertebrobasilar dolichoectasia; IADE, intracranial arterial dolichoectasia; TCV, total cranial volume*.

Due to the association of IADE with lacunar infarctions, the prevalence of abnormally dilated arteries may vary according to stroke mechanisms. The occurrence of dolichoectasia is greater when infarctions in the posterior circulation are exclusively considered (30, 32). Conversely, the study of Nakamura and colleagues found higher prevalence of dolichoectasia among patients with brain hemorrhages when compared to those with ischemic injuries (12 vs 6%) (40).

## Risk Factors

Arterial dolichoectasia seems to be related to hemodynamic and anatomical factors in the setting of individual-to-individual susceptibility for developing this arteriopathy. Across series, aging, male gender, and arterial hypertension have been consistently associated with IADE (6, 7, 10). Other traditional factors such as cigarette smoking, increased body-mass index, dyslipidemia, and diabetes mellitus showed conflicting results (6, 14). Due to similar risk factors, it has been postulated that IADE and atherosclerosis shared common pathogenesis. The presence of atheromatous plaque associated with IADE seems to happen exclusively in the dolichoectatic vessel and is thought to be a consequence rather than the cause of abnormal blood flow. Conversely, the association of IADE with aortic aneurysm, coronary ectasia, and kinked carotid arteries argues in favor to a generalized susceptibility for arterial dilation.

Wolfe et al. showed increased occurrence among African-American patients (36). A study involving a large series of patients with lacunar infarctions demonstrated that white race was independently associated with VBD (14). A multiethnic cohort of a predominantly Hispanic population found a higher prevalence of IADE compared to other reports. However, an ethnic association with IADE has not been fully established (6).

A variety of hereditary conditions including Marfan syndrome, Ehlers–Danlos syndrome type IV, pseudoxanthoma elasticum, neurofibromatosis type 1, tuberous sclerosis complex, Fabry disease, Pompe disease, moyamoya disease, cavernous malformations, autosomal dominant polycystic kidney disease, and the acquired immune deficiency syndrome, have been associated with IADE (43–53).

## IADE and SVD

Results from two case-control studies found that individuals with stroke and IADE had a higher prevalence of lacunar infarctions (36–42%) compared to those without IADE (17–19%) (7, 41). In one of these studies, small artery occlusion was the only stroke mechanism associated with IADE (OR 2.89; C.I. 1.29–6.46) (7). When compared with patients without IADE, the same group of investigators found that patients with IADE and ischemic stroke have a higher prevalence of multilacunar state, severe leukoaraiosis, and enlarged perivascular spaces (20). Cerebral microbleeds, another imaging marker of SVD, were more prevalent in stroke patients with IADE than in those without (39). Furthermore, the autopsy study established an association between IADE and sclerosis and hyalinosis of small penetrating arteries (21).

These findings suggest an underlying common physiopathology for IADE and SVD. Abnormal activity of the matrix metalloproteinase family (MMP) has been hypothesized as a potential bridge mechanism between these two conditions (54). MMPs are a family of proteases that act over the extracellular matrix as the rate-limiting step for connective tissue remodeling (55). At the vascular level, MMPs with elastase and collagenase activity (i.e., MMP-2, MMP-3, and MMP-9) have shown to be molecular contributors to aneurysm formation as well as instability of atherosclerotic plaques (55–58). The MMP-3 5A allele, which is associated with an increased proteolytic activity, is found in higher frequency in patients with abdominal aortic aneurysms and coronary artery aneurysms (57, 59, 60). Pico et al. showed similar results for the MMP-3 5A genotype associated with IADE, implying a role of MMPs in the development of cerebral dilative arteriopathy (54). Conversely, abnormal upregulation of MMPs at the cellular level promote cytotoxicity and central nervous system inflammation by degradation of basal lamina proteins leading to disruption of the blood–brain barrier and breakdown of myelin (61–63). Increased MMPs activity is linked to the pathogenesis of several central nervous system diseases including multiple sclerosis, Alzheimer’s disease, and acute stroke (64, 65). Among patients with SVD, pathological studies have shown diffuse inflammatory response associated with high levels of MMP-3-positive macrophages in the regions of white matter damage as well as clustering of cells expressing MMP-2 and MMP-3 around the small penetrating vessels (66).

Different physiological and pathological conditions upregulate the expression of MMPs (63). IADE and SVD might have common triggers that activate a cascade of events that lead to an abnormal proteolytic balance turning over in the pathological changes and clinical consequences characteristics of these conditions. These enzymes might represent a potential therapeutic target to modify the natural course of these two cerebrovascular diseases.

## Clinical Course, Prognosis, and Treatment

Intracranial arterial dolichoectasia can be asymptomatic or may become complicated by one of the following mechanisms: (1) abnormal antegrade laminar blood flow predisposing to thrombi formation with or without embolic phenomena (33, 67); (2) occlusion of small penetrating vessels either by stretching and obliteration of arteries raising from the affected vessel or by its association with SVD (7, 20, 41, 68); (3) compression of surrounding structures including brainstem and cranial nerves (35, 69–73); (4) CSF flow obstruction with resultant hydrocephalus either by direct compression of the third ventricle or by transmission of pulsations causing a “water-hammer” effect (74, 75); (5) vessel wall rupture with catastrophic brain hemorrhages (17, 76, 77). A systematic review including a total of 375 patients with VBD determined the 5-year risk rate for brain infarction (17.6%), brainstem compression (10.3%), transient ischemic attack (10.1%), hemorrhagic stroke (4.7%), hydrocephalus (3.3%), and subarachnoid hemorrhage (2.6%) (9). The same review reported a 5-year mortality risk of 36.2%, with ischemic stroke as the most common cause of death (9). Factors associated with adverse clinical outcome include symptoms at the time of diagnosis, severity of arterial dilation and dolichosis, mural T1 signal, mural thrombi, and interval ectasia progression on follow-up neuroimaging (9–11, 13, 36, 78).

Information on the appropriate management of patients with IADE is scarce. Patients presenting with compressive neurological manifestations should probably have surgical evaluation. Cranial nerve surgical decompression can be achieved by repositioning of the artery (79), while hydrocephalus due to IADE is usually refractory to ventricular shunt placement (8). For patients with cerebrovascular complications, acute management should be based on best care practices for patients with hemorrhagic or ischemic strokes, as no controlled clinical trials have studied the effectiveness of secondary prevention for patients with IADE and stroke (80, 81). Anticoagulant use is controversial (82) and might represent additional risk for hemorrhagic complications (17, 76). Due to the common coexistence with SVD, vascular risk factors control and the use of antiplatelet therapy and statins are advised (3).

Among individuals with newly diagnosed IADE, it is recommended to screen for other potentially fatal arterial disorders including intracranial saccular aneurysms, abdominal aortic aneurysms, and coronary artery disease (3, 11, 18, 19). In young subjects and in those without vascular risk factors, primary predisposing conditions should be considered (i.e., Marfan’s, Fabry’s, adult-onset Pompe’s disease, etc.). Serial imaging at 6 months and then yearly to monitor for arterial enlargement is advised (3). There is no validated stratification according to severity for IADE. Patients at high risk of fatal complications based on severe arterial dilation (>10 mm) or interval arterial enlargement after diagnosis (>2 mm increase in follow-up imaging) should avoid anticoagulation and have strict blood pressure control (3, 17). This subgroup of patients might benefit from surgical repair of the vascular defect. Good outcomes have been reported in studies focused on patients with gigantic dilations (83). Either vessel reconstruction by open surgery (83, 84) or endovascular coiling and/or stenting (85) aim to eliminate the risk of bleeding or mass effect without compromising vessel patency. The technical approach is usually complex and needs to be tailored to each case.

## Conclusion

Intracranial arterial dolichoectasia implies a spectrum that includes normal variations in the diameter and tortuosity of the cerebral vasculature as well as gigantic aneurysmal dilations with a high risk of fatal complications. In addition, IADE may involve one or more arteries or may present as a dynamic progressive arteriopathy. All of these factors add to the risk of neurological complications, with ischemic stroke as the most common one. Proposed diagnostic criteria and severity grading based on non-invasive and cost-effective diagnostic tests need to be validated across populations to elucidate the clinical significance of this vasculopathy.

## Author Contributions

VB and JO contributed to writing of the manuscript and edited the tables and figures. JB contributed to writing and editing of the manuscript, tables, and figures.

## Conflict of Interest Statement

The authors declare that the research was conducted in the absence of any commercial or financial relationships that could be construed as a potential conflict of interest.
